# Personalized management of platelet transfusion refractoriness: risk factors, cross-matching, and IVIG strategies

**DOI:** 10.3389/fmed.2026.1923303

**Published:** 2026-09-18

**Authors:** Jixin Li, Linting Wang, Yi Qin, Yingyin Liang, Piao Lv, Yuan Yao, Xinxin Fan, Huayou Zhou

**Affiliations:** Department of Transfusion Medicine, Nanfang Hospital, Southern Medical University, Guangzhou, Guangdong, China

**Keywords:** alloimmunization, corrected count increment (CCI), hematologic disorders, intravenous immunoglobulin (IVIG), platelet antibodies, platelet cross-matching, platelet transfusion refractoriness, risk factors

## Abstract

**Background:**

Platelet transfusion refractoriness (PTR) is a major clinical challenge in hematologic patients, yet real-world evidence comparing transfusion strategies—particularly the role of cross-matched platelets and intravenous immunoglobulin (IVIG)—remains limited.

**Methods:**

We retrospectively analyzed 144 hematologic patients diagnosed with PTR at Nanfang Hospital (2019–2022), totalling 1,345 transfusion episodes. Patients were stratified by platelet antibody status. Four transfusion regimens were compared: random platelets, cross-matched platelets, random platelets + IVIG, and cross-matched platelets + IVIG. The primary endpoint was 24-h corrected count increment (CCI). Logistic regression identified factors associated with antibody development. The primary analysis was performed using linear mixed-effects models (LMMs) with a random intercept for patients to account for within-patient correlation. Fixed effects included transfusion strategy along with prespecified episode-level and patient-level covariates. Generalized estimating equations (GEE) were used as a sensitivity analysis to assess the robustness of the findings.

**Results:**

Female sex emerged as the strongest independent predictor for the development of platelet antibodies (OR: 4.18, 95% CI: 2.25–8.24). Other univariate risk factors included age, obstetric history, diagnosis, and chemotherapy cycles. Among antibody-positive patients, both cross-matched platelets and IVIG combined with random platelets were associated with improved 24 h CCI compared with random platelets alone (all *p*_Holm_ < 0.001), cross-matched platelets were associated with higher 24 h CCI relative to random plus IVIG (*p*_Holm_ = 0.019), whereas cross-matched platelets plus IVIG demonstrated no additional benefit vs. cross-matched platelets alone in this cohort (*p*_Holm_ = 0.444). In antibody-negative patients, no statistically significant difference in 24 h CCI was observed across the four transfusion strategies (*p* = 0.244). Consistent with the LMM results, the overall effect of transfusion strategy was not statistically significant among antibody-negative patients (*p* = 0.692) but was significant among antibody-positive patients (*p* < 0.001). Similar results were obtained using GEE, supporting the robustness of these findings.

**Conclusion:**

Among patients with antibody-negative PTR, cross-matched platelets and IVIG demonstrated no additional benefit in this cohort. For antibody-positive PTR, cross-matched platelets showed the most favorable response in this cohort; when unavailable, high-dose IVIG with random platelets may be preferentially considered. These comparative treatment findings require prospective validation.

## 1 Introduction

Platelet transfusions are essential for patients with cancer, hematologic malignancies, bone marrow failure, and recipients of hematopoietic stem cell transplantation—they prevent severe bleeding (1, 2). But PTR—failure to achieve expected posttransfusion increments—can complicate care and threaten survival (3, 4). Reported PTR incidence in hematologic patients ranges from 7% to 34%, depending on the population and definition (5–7). The standard definition requires two consecutive transfusions of ABO-identical, fresh platelets without the expected rise in platelet count or clinical improvement (8–10).

Management strategies for PTR depend on the underlying cause, especially the presence of platelet antibodies (11–13). For immune-mediated PTR, guidelines recommend crossmatch-compatible or HLA-selected platelets. For non-immune causes, treatment targets the primary condition. But how best to diagnose immune PTR (IPTR) is still unclear (14). Solid-phase red cell adherence assay (SPRCA) and other methods can miss low-titer or low-affinity antibodies, so clinically relevant alloimmunization may go undetected.

On the other hand, for antibody-negative patients in whom non-immune causes are not obvious, evidence to guide platelet selection is limited. Our study may help reduce unnecessary use of cross-matched platelet products and associated resource use.

Other immune thrombocytopenia (ITP) therapies, particularly IVIG, have been used for immune PTR (11), but evidence for benefit is conflicting (15–17). We therefore asked: what factors predict platelet antibody development in hematologic patients with PTR? And how well do cross-matched platelets with or without IVIG perform in these patients? Our findings can help identify high-risk patients for alloimmunization, guide rational use of cross-matched platelets and IVIG, and improve resource allocation.

## 2 Materials and methods

### 2.1 Study design and patients

We retrospectively analyzed patients with hematologic disorders diagnosed with platelet transfusion refractoriness (PTR) at the Department of Hematology, Nanfang Hospital, Southern Medical University, between January 2019 and June 2022. PTR was defined as meeting at least one of the following criteria after two consecutive ABO-identical random apheresis platelet transfusions: 1-h corrected count increment (CCI) < 7.5, 24-h CCI < 4.5 (18).

Inclusion criteria were: (1) diagnosis of a hematologic disorder; (2) meeting PTR criteria; and (3) complete clinical data, including age, sex, ABO blood type, height, weight, disease diagnosis, obstetric history, treatment history, platelet transfusion details (time, dose, complete blood count within 24 h before and after transfusion), body temperature, and IVIG infusion data. Exclusion criteria were: (1) no platelet transfusion; or (2) non-refractory response.

### 2.2 Grouping

Patients were stratified by platelet antibody status (positive vs. negative); by age (≤30, 31–50, >50 years); by sex; by obstetric history (women only); by ABO type; by diagnosis [acute/chronic myeloid leukemia (AML/CML), acute/chronic lymphoblastic leukemia (ALL/CLL), myelodysplastic syndrome (MDS), lymphoma, other]; and by cumulative platelet transfusions (1–3, 4–6, 7–9, ≥10). Transfusion episodes were categorized into four regimens: random platelets, cross-matched platelets, random platelets plus IVIG, and cross-matched platelets plus IVIG.

### 2.3 Platelet transfusion

Indications followed published guidelines (11, 12, 19): prophylactic transfusion when platelet count <10 × 10^9^/L without bleeding; when <20 × 10^9^/L with bleeding risk; and higher thresholds (50 × 10^9^/L) for active bleeding, surgery, or invasive procedures. Red cells and plasma were given per standard indications.

Apheresis platelets were from Guangzhou Blood Center, ABO- and Rh-matched, prepared to national quality standards (≥2.5 × 10^11^ platelets per unit), stored at 22 ± 2 °C with agitation, and used within expiry.

### 2.4 IVIG administration

IVIG (5–25 g) was infused intravenously within 24 h before platelet transfusion, over 2 h.

### 2.5 Outcome measures

We used 24-h CCI as the primary efficacy metric (18, 20), calculated as:

$$\begin{array}{rl} \mathrm{CCI} & =(\mathrm{post}\text{-}\mathrm{transfusion}\;\mathrm{platelet}\;\mathrm{count}\text{–}\mathrm{pre}\text{-}\mathrm{transfusion}\;\mathrm{platelet}\;\mathrm{count})\\ & \times \mathrm{body}\;\mathrm{surface}\;\mathrm{area}(m^{2})/\mathrm{number}\;\mathrm{of}\;\mathrm{platelets}\;\mathrm{transfused}(10^{11}) \end{array}$$

### 2.6 Data collection

Clinical data were extracted from the hospital's electronic medical records, including admission and discharge notes, nursing records, laboratory results, and examinations. Baseline data were collected at the time of first PTR diagnosis.

Collected variables included: age, sex, ABO type, height, weight, diagnosis, obstetric history, treatment history, outcome; platelet transfusion details (time, cumulative number, dose, platelet counts within 24 h before/after transfusion, body temperature); and clinical conditions at transfusion [infection, fever, sepsis, splenomegaly, disseminated intravascular coagulation (DIC), graft-versus-host disease (GVHD)].

### 2.7 Platelet antibody detection

Anti-HLA class I and anti-HPA antibodies [against GP Ib/IX, GP IIb/IIIa, GP Ia/IIa, and GP IV (CD36)] were detected using solid-phase enzyme-linked immunosorbent assay (Lifecodes Pakplus, Immucor, USA). Plates were read on an EL × 800GTI microplate reader (BioTek). A sample was considered positive when the optical density was ≥2-fold the negative control.

### 2.8 Platelet cross-matching

Solid-phase red cell adherence assay (SPRCA) was used (MASPAT, Sanquin, the Netherlands). Donor platelets were immobilized on microplate wells pre-coated with murine monoclonal anti-platelet antibodies. Recipient serum was added with low-ionic-strength solution. Bound antibodies were detected with anti-human IgG and MASPAT indicator red cells. Positive reactions (presence of platelet antibodies) showed a uniform layer of red cells; negative reactions showed a compact button.

### 2.9 Statistical analysis

The dataset had a two-level hierarchical structure, with platelet transfusion episodes (level 1) nested within patients (level 2). Analyses were performed separately in the platelet antibody–negative and platelet antibody–positive cohorts.

In the antibody-negative cohort, 710 episodes were contributed by 83 patients (median 7 episodes per patient, IQR: 4–12, range: 2–27); in the antibody-positive cohort, 635 episodes were contributed by 61 patients (median: 9, IQR: 6–13, range: 3–39). Importantly, every patient in both cohorts received at least two of the four transfusion strategies (antibody-negative: 6.0% two strategies, 63.9% three, 30.1% four; antibody-positive: 1.6%, 67.2% and 31.1%, respectively), so that the design was fully connected and partially crossed rather than nested, and all between-strategy contrasts were informed by within-patient comparisons.

Handling of repeated episodes at each stage of analysis. Repeated measurements within patients were addressed explicitly at every analytical stage, as follows.

- Episode-level 24 h CCI values were first averaged within each patient for each transfusion strategy, yielding one value per patient per strategy; violin plots and group summaries were then constructed from these patient-level means. No descriptive comparison was performed at the episode level.
- For each pair of strategies, the analysis was restricted to patients who had received both strategies, and the patient-level means were compared with the Wilcoxon signed-rank test. The number of patients available for each pairwise contrast therefore differs (antibody-positive cohort: *n* = 20–59; antibody-negative cohort: *n* = 26–77) and is smaller than the number of episodes. *p* values were additionally adjusted for the six pairwise comparisons using the Holm procedure.
- Omnibus comparison across the four strategies. Because the four strategy groups shared virtually all patients, an unpaired omnibus test would have been invalid. The four strategies were therefore compared using the Skillings-Mack test, with patients as blocks and patient-level mean CCI as the response.
- Primary analysis (all episodes). All 1,345 episodes (635 antibody-positive, 710 antibody-negative) were analyzed with linear mixed-effects models (LMM) including a random intercept for patient. Fixed effects comprised transfusion strategy (reference: random platelets without IVIG) and pre-specified covariates: episode-level (time-varying) covariates—fever (body temperature ≥38 ℃) and transfusion sequence number; patient-level covariates—sex, age category, diagnostic group, treatment history, number of chemotherapy cycles, splenomegaly, GVHD, infection, DIC, and the cumulative number of prior platelet transfusions. Models were fitted by restricted maximum likelihood; denominator degrees of freedom were obtained by the Satterthwaite approximation. Between-strategy differences were expressed as estimated marginal means (EMM) and adjusted mean differences with 95% confidence intervals, and all six pairwise contrasts were adjusted using Tukey's method. Multicollinearity was assessed by generalised variance-inflation factors. A model with random slopes for strategy was also examined but was not retained (singular fit/no improvement by likelihood-ratio test).
- Quantification of clustering. The intraclass correlation coefficient (ICC) was calculated from an intercept-only model, and the significance of the patient random intercept was tested by likelihood-ratio test. The conventional design effect was computed for reference; because transfusion strategy varied within patients, the design effect relevant to the strategy contrasts approximates 1-ICC, so that accounting for clustering increases rather than decreases the precision of within-patient contrasts.
- Decomposition of within- and between-patient effects. To evaluate potential confounding by indication, the strategy effect was decomposed into within- and between-patient components using cluster-mean centring, and the equality of the two components was tested with a joint Wald test on the contextual (Mundlak) terms.
- Marginal (population-averaged) models. As an alternative to the LMM, generalised estimating equations (GEE) with an identity link, Gaussian variance function, exchangeable working correlation and robust (sandwich) standard errors clustered on patient were fitted with the same covariates; independence and AR(1) working correlations were examined as alternatives and compared using QIC. With 61 and 83 clusters, the number of clusters was sufficient for reliable sandwich variance estimation.

Sensitivity analyses. The following pre-specified sensitivity analyses were performed: (a) unadjusted LMM; (b) LMM restricted to the first episode of each strategy per patient; (c) exclusion of patients contributing more than the 95th percentile of episodes; (d) rank-based (van der Waerden inverse-normal) LMM, given the right-skewed distribution of CCI (skewness 1.56 and 1.06); (e) LMM allowing heteroscedastic residual variance across strategies; and (f) GEE with alternative working correlation structures. For reference, a naïve episode-level analysis treating all episodes as independent (one-way ANOVA with Tukey correction and Kruskal–Wallis test) is also reported, to quantify the impact of ignoring clustering.

Normality was checked with Shapiro–Wilk test. Normally distributed data are shown as mean ± SD; two-group comparisons used *t* tests. Categorical data are presented as *n* (%) and compared by chi-square test. Unconditional logistic regression (univariate and multivariate) was used to calculate odds ratios (OR) and 95% confidence intervals (CI) based on Wald test. Regardless of statistical significance, all pairwise comparisons consistently report Cohen's *d_z_* as the standardized effect size. Cohen's *d_z_* was calculated as the mean of patient-level difference scores divided by the standard deviation of difference scores between two transfusion strategies in the same patient.

$$d_{z}=\frac{\bar{D}}{SD_{D}}(D=X_{1}-X_{2})$$

Effect sizes: small 0.2 ≤ *d_z_* < 0.5, medium 0.5 ≤ *d_z_* < 0.8, large *d_z_* ≥ 0.8.

All analyses were performed in R version 4.4.1 (R Foundation for Statistical Computing, Vienna, Austria) using the lme4, lmerTest, emmeans and geepack packages. Two-sided *p* < 0.05 was considered statistically significant.

### 2.10 Ethics

The retrospective protocol of this study was approved by the Ethics Committee of the Nanfang Hospital, Southern Medical University (Ethical approval number: NFEC-2018-143) prior to initiation of study, and the requirement for informed consent was waived. Originally granted in 2018 as a prospective approval for an ongoing clinical database, this ethics approval formally covers the entire patient-enrolment period from January 2019 to June 2022; no separate renewal or extension was required.

## 3 Results

### 3.1 Patient demographics and baseline characteristics

We included 144 patients with hematologic disorders diagnosed with PTR at the Nanfang Hospital between January 2019 and June 2022. A total of 1,345 platelet transfusion episodes were analyzed, the distribution of which by patient and strategy, and the number of patients shared by each pair of strategies, are given in Supplementary Tables S1 and S2.

The 1,345 transfusion episodes were contributed by 144 patients (83 antibody-negative and 61 antibody-positive). All patients received more than one transfusion strategy during the study period (Supplementary Table S1), and each pair of strategies was shared by a substantial number of patients (20–77; Supplementary Table S2), indicating a fully connected, partially crossed design. Within-patient clustering of 24-h CCI was statistically significant in both cohorts (likelihood-ratio test for the patient random intercept: *χ*^2^ = 32.06, *p* < 0.001 in antibody-negative patients; *χ*^2^ = 13.38, *p* < 0.001 in antibody-positive patients). The ICC was 0.186 in antibody-negative patients and 0.124 in antibody-positive patients; that is, 12%–19% of the total variability in 24 h CCI was attributable to stable between-patient differences. With mean cluster sizes of 8.6 and 10.4 episodes, the conventional design effects were 2.41 and 2.17, corresponding to effective sample sizes of approximately 295 and 293 independent observations rather than 710 and 635, and to an inflation of standard errors by factors of 1.55 and 1.47 had clustering been ignored for patient-level comparisons.

Mean patient age was 42.38 ± 13.79 years; 68 patients (47.22%) were male, 76 patients (52.78%) were female. Platelet antibodies were positive in 61 patients (42.36%) and negative in 83 (57.64%). Diagnoses included acute/chronic myeloid leukemia (AML/CML, *n* = 72, 50.00%), acute/chronic lymphoblastic leukemia (ALL/CLL, *n* = 33, 22.92%), myelodysplastic syndrome (MDS, *n* = 20, 13.89%), lymphoma (*n* = 7, 4.86%), and other hematologic diseases (*n* = 12, 8.33%). Baseline clinical covariates (splenomegaly, GVHD, infection, sepsis, and DIC) were comparable between the antibody-negative and antibody-positive groups, with no statistically significant inter-group differences. Results are summarized in Table 1.

**Table 1:** Demographics and baseline characteristics of patients.

Clinical characteristics	Group	Negative group	Positive group	*p*
		*n* = 83	*n* = 61	
Gender (%)				<0.001
	Male	55 (66.3)	13 (21.3)	
	Female	28 (33.7)	48 (78.7)	
Age, years (mean ± SD)		40.2 ± 14.6	45.3 ± 12.2	0.029
Blood Groups, *n* (%)				0.639
	A	22 (26.5)	17 (27.9)	
	B	27 (32.5)	14 (23.0)	
	O	25 (30.1)	22 (36.1)	
	AB	9 (10.8)	8 (13.1)	
Pregnancy history				0.047
(among female patients), *n* (%)				
	No	3 (10.7)	0 (0.0)	
	Yes	25 (89.3)	48 (100.0)	
Disease type, *n* (%)				0.002
	AML/CML	35 (42.2)	37 (60.7)	
	ALL/CLL	25 (30.1)	8 (13.1)	
	MDS	7 (8.4)	13 (21.3)	
	Lymphoma	6 (7.2)	1 (1.6)	
	Other*	10 (12.0)	2 (3.3)	
Treatment history, *n* (%)				0.022
	Chemotherapy	31 (37.3)	37 (60.7)	
	HSCT	37 (44.6)	17 (27.9)	
	Other	15 (18.1)	7 (11.5)	
Splenomegaly, *n* (%)				0.112
	No	30 (36.1)	31 (50.8)	
	Yes	53 (63.9)	30 (49.2)	
GVHD, *n* (%)				0.073
	No	66 (79.5)	56 (91.8)	
	Yes	17 (20.5)	5 (8.2)	
Infection, *n* (%)				0.355
	No	76 (91.6)	52 (85.2)	
	Yes	7 (8.4)	9 (14.8)	
Sepsis, *n* (%)				0.355
	No	76 (91.6)	52 (85.2)	
	Yes	7 (8.4)	9 (14.8)	
DIC, *n* (%)				0.637
	No	80 (96.4)	60 (98.4)	
	Yes	3 (3.6)	1 (1.6)	
Clinical outcome, *n* (%)				0.120
	Improved	60 (72.3)	53 (86.9)	
	Deteriorated	16 (19.3)	6 (9.8)	
	Death	7 (8.4)	2 (3.3)	

AML, acute myeloid leukemia; CML, chronic myeloid leukemia; ALL, acute lymphoblastic leukemia; CLL, chronic lymphocytic leukemia; MDS, myelodysplastic syndromes.

* Other diagnoses included: Acute mixed phenotype leukemia, B-lymphoblastic leukemia/lymphoma, Myeloid/lymphoid neoplasms with PDGFRB rearrangement, Multiple myeloma, Waldenström macroglobulinemia, Immune thrombocytopenia (ITP), and Primary myelofibrosis; HSCT, Hematopoietic stem-cell transplantation; GVHD, Graft-versus-host disease; DIC, Disseminated intravascular coagulation.

Among 61 antibody-positive patients, 40 patients (65.57%) had HLA antibodies alone, 8 (13.11%) HPA antibodies alone, 10 (16.39%) both HLA and HPA antibodies, and 3 (4.92%) HLA, HPA, and CD36 antibodies. Overall, HLA antibodies were detected in 53 of 61 positive patients (86.89%), HPA antibodies in 21 (34.43%), and CD36 antibodies in 3 (4.92%). In the entire cohort of 144 PTR patients, the prevalence was 36.81% for HLA antibodies (53/144), 14.58% for HPA antibodies (21/144), and 2.08% for CD36 antibodies (3/144).

### 3.2 Factors associated with platelet antibody development

#### 3.2.1 Univariate logistic regression

We examined age, sex, blood type, diagnosis, obstetric history, cumulative platelet transfusions, and number of chemotherapy cycles as independent variables, with antibody status as the dependent variable. ABO blood type (*p* = 0.639) and cumulative platelet transfusions (*p* = 0.705) showed no significant association. Significant associations were found for sex (*p* < 0.001), age (*p* = 0.039), diagnosis (*p* = 0.002), number of chemotherapy cycles (*p* < 0.001), and obstetric history in women (*p* = 0.047).

Women had a 7.09-fold higher risk of developing antibodies than men (OR: 7.09, 95% CI: 3.37–15.80). Using patients aged ≤30 years as the reference group, patients aged 31–50 years had a 2.98-fold higher risk of antibody development (OR: 2.98, 95% CI: 1.19–8.10), and those aged >50 years had a 2.93-fold higher risk (OR: 2.93, 95% CI: 1.14–8.19). Risk of platelet antibody development was highest during the first chemotherapy cycle. Using ≤1 chemotherapy cycle as the reference group, patients who received 2–4 cycles had a 71% lower risk of antibody development (OR: 0.29, 95% CI: 0.12–0.65) and those with ≥5 cycles had a 78% lower risk (OR: 0.22, 95% CI: 0.09–0.51). Compared with patients with other hematologic disorders, those with MDS had an 8.24-fold higher risk of antibody development (OR: 8.24, 95% CI: 1.57–71.70), and patients with AML/CML exhibited a 4.92-fold elevated risk (OR: 4.92, 95% CI: 1.17–37.00). Among women, those with a history of pregnancy had higher antibody positivity rates than those without. Results are summarized in Table 2.

**Table 2:** Univariate and multivariate logistic regression analysis.

Factors	Univariate Logistic regression analysis				Multivariate Logistic regression analysis	
	Negative group (*n* = 83)	Positive group (*n* = 61)	OR (95%CI)	*p*	OR (95%Cl)	*p*
Age				0.039		
≤30 y	26 (31.3)	8 (13.1)	Ref.		Ref.	
31–50 y	31 (37.3)	29 (47.5)	2.98 (1.19, 8.10)	0.019	1.50 (0.62, 3.74)	0.4
>50 y	26 (31.3)	24 (39.3)	2.93 (1.14, 8.19)	0.025	1.28 (0.62, 2.74)	0.5
Gender				<0.001		
Male	55 (66.3)	13 (21.3)	Ref.		Ref.	
Female	28 (33.7)	48 (78.7)	7.09 (3.37, 15.80)	<0.001	4.18 (2.25, 8.24)	<0.001
Pregnancy history (among female patients)				0.047		
No (*n* = 3)	3 (10.7)	0 (0.0)	—			
Yes (*n* = 73)	25 (89.3)	48 (100.0)	—			
Blood Groups				0.639		
A	22 (26.5)	17 (27.9)	Ref.			
B	27 (32.5)	14 (23.0)	0.68 (0.27, 1.68)	0.399		
O	25 (30.1)	22 (36.1)	1.14 (0.48, 2.71)	0.771		
AB	9 (10.8)	8 (13.1)	1.15 (0.35, 3.70)	0.816		
Disease type				0.002		
Other*	10 (12.0)	2 (3.3)	Ref.		Ref.	
MDS	7 (8.4)	13 (21.3)	8.24 (1.57, 71.70)	0.011	0.97 (0.26, 4.95)	>0.9
AML/CML	35 (42.2)	37 (60.7)	4.92 (1.17, 37.00)	0.028	0.79 (0.10, 3.59)	0.8
ALL/CLL	25 (30.1)	8 (13.1)	1.52 (0.30, 12.60)	0.635	1.92 (0.43, 8.35)	0.4
Lymphoma	6 (7.2)	1 (1.6)	0.88 (0.02, 13.20)	0.931	9.86 (2.20, 57.0)	0.005
PLT transfusion numbers				0.705		
1–3	6 (7.2)	2 (3.3)	Ref.		Ref.	
4–6	16 (19.3)	15 (24.6)	2.64 (0.49, 22.40)	0.270	0.70 (0.17, 3.32)	0.6
7–9	14 (16.9)	9 (14.8)	1.83 (0.32, 16.20)	0.518	0.91 (0.24, 3.15)	0.9
≥10	47 (56.6)	35 (57.4)	2.12 (0.44, 16.80)	0.367	1.06 (0.36, 3.17)	>0.9
Chemotherapy cycles				<0.001		
≤1	24 (28.9)	38 (62.3)	Ref.		Ref.	
2–4	29 (34.9)	13 (21.3)	0.29 (0.12, 0.65)	0.003	0.68 (0.28, 1.66)	0.4
≥5	30 (36.1)	10 (16.4)	0.22 (0.09, 0.51)	<0.001	1.65 (0.70, 3.98)	0.3

OR, odds ratio; CI, confidence interval; Ref, reference group; PLT, platelet; AML, acute myeloid leukemia; CML, chronic myeloid leukemia; ALL, acute lymphoblastic leukemia; CLL, chronic lymphocytic leukemia; MDS, myelodysplastic syndromes.

* Other diagnoses included: Acute mixed phenotype leukemia, B-lymphoblastic leukemia/lymphoma, Myeloid/lymphoid neoplasms with PDGFRB rearrangement, Multiple myeloma, Waldenström macroglobulinemia, Immune thrombocytopenia (ITP), and Primary myelofibrosis; “—”: Odds ratios could not be calculated for categories with zero cell counts. Univariate analysis was performed using logistic regression, and variables with *p* < 0.05 in the univariate analysis were included in the multivariate logistic regression model.

#### 3.2.2 Multivariate logistic regression

Multicollinearity among independent variables was assessed using the variance inflation factor (VIF). Variables with a VIF > 5 were excluded from the final multivariate logistic regression model. Owing to substantial multicollinearity between obstetric history and sex, obstetric history was removed, and only sex was retained in the model. After adjusting for covariates, sex remained an independent risk factor for platelet antibody development (*p* < 0.001). Women had a 4.18-fold higher risk of developing antibodies than men (OR: 4.18, 95% CI: 2.25–8.24). In subgroup analyses stratified by diagnosis, the lymphoma subgroup was based on only seven patients with a single antibody-positive event. Owing to the small sample size, the confidence interval was wide and unstable. The corresponding OR value (OR: 9.86, 95% CI: 2.20–57.00) should therefore be interpreted with extreme caution. Age and number of chemotherapy cycles, although significant in univariate analysis, were not independent predictors after multivariate adjustment (all *p* > 0.05). Results are summarized in Table 2.

### 3.3 Efficacy of different transfusion strategies in antibody-negative patients with PTR

#### 3.3.1 Omnibus comparison across the four transfusion strategies

Among patients with antibody-negative, the 24 h CCI (mean ± SD) for random platelets was 4.15 ± 5.56, for cross-matched platelets 5.33 ± 5.95, for random platelets + IVIG 3.75 ± 4.36, and for cross-matched platelets + IVIG 4.80 ± 6.18. The Skillings-Mack test across the four strategies, which accounts for the incomplete-block structure, showed no overall difference (*SM* = 4.16, *df* = 3, *p* = 0.244; Figure 1A).

**Figure 1:**
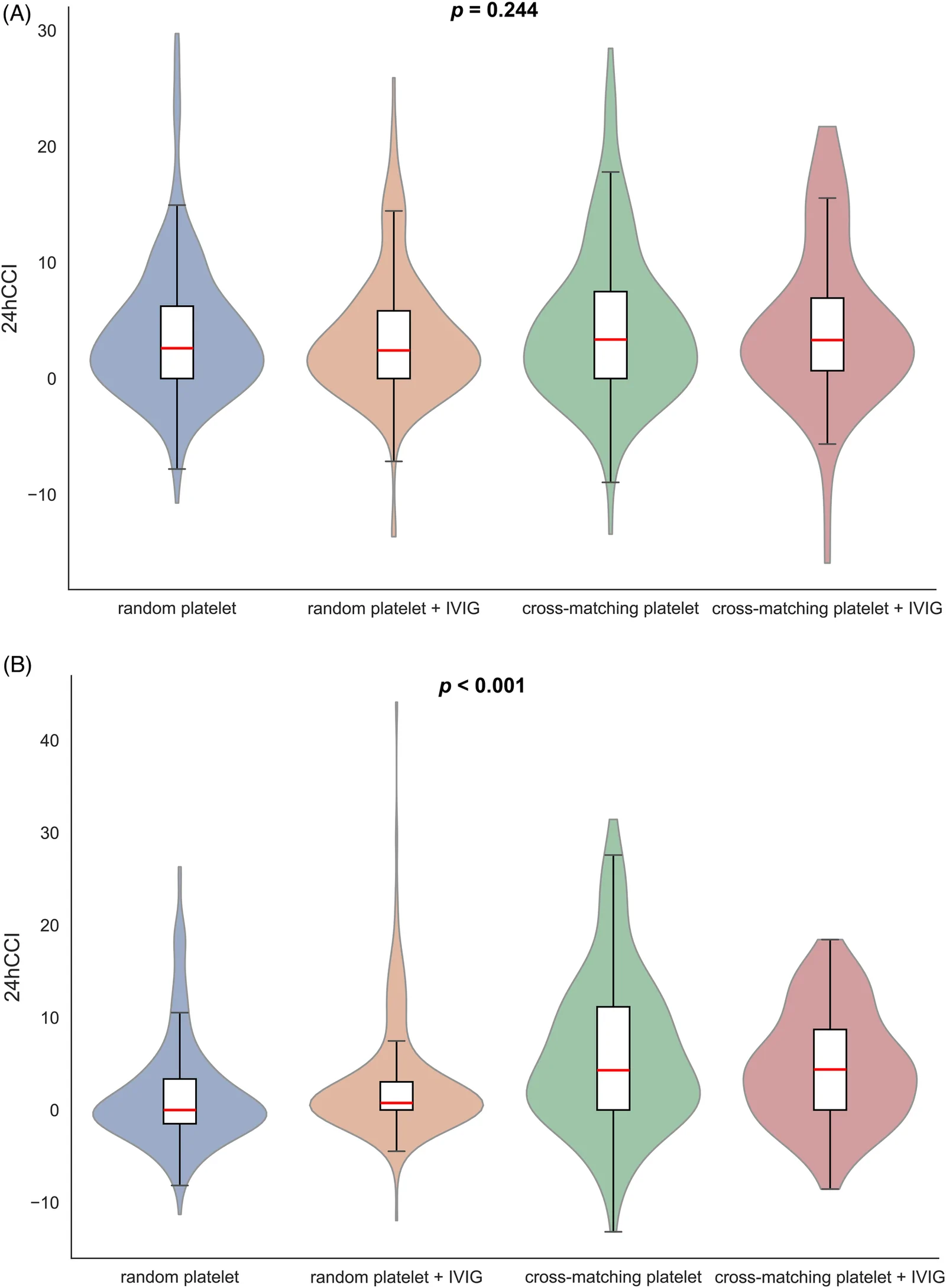
Comparison of treatment responses to four platelet transfusion regimens in antibody-negative and -positive patients with PTR. The four transfusion regimens in platelet antibody-negative patients **(A)** and antibody-positive patients **(B)** were: random platelets, random platelets + IVIG, cross-matching platelets, and cross-matching platelets + IVIG.

#### 3.3.2 Pairwise comparisons between transfusion strategies

For each pairwise comparison of transfusion strategies, the analysis was restricted to patients receiving both interventions. Patient-level mean 24 h CCI values were compared using the Wilcoxon signed-rank test. Raw *p* values were adjusted via the Holm procedure to account for all six possible pairwise comparisons across the four strategies. Nevertheless, only four prespecified clinically relevant contrasts are presented in the results for this cohort. Specifically:

- Random + IVIG vs. random alone: *p*_Holm_ = 1.00, Cohen's *d_z_* = 0.06 (small effect).
- Cross-matched vs. random platelets: *p*_Holm_ = 0.66, Cohen's *d_z_* = 0.17 (small effect).
- Cross-matched + IVIG vs. random platelets: *p*_Holm_ = 1.00, Cohen's *d_z_* = 0.03 (small effect).
- Cross-matched + IVIG vs. cross-matched alone: *p*_Holm_ = 1.00, Cohen's *d_z_* = 0.01 (small effect).

After Holm correction, Wilcoxon signed-rank tests revealed no significant differences between any two regimens (all *p*_Holm_ > 0.05). Accordingly, among patients with antibody-negative PTR, cross-matched platelets and IVIG were not associated with improved 24-h CCI (Table 3).

**Table 3:** Pairwise comparisons between transfusion strategies in antibody-negative patients with PTR.

Paired group (*x*_1_ vs. *x*_2_)	*n*	Median (Q1–Q3)		Median difference	*Wilcoxon W*	*z*	*p_raw_*	*p*_Holm_	Cohen's *d_z_*
		*x*_1_	*x*_2_						
Random platelets vs. Random platelets + IVIG	77	3.04 (0.77–4.93)	2.72 (1.09–5.40)	0.32	1,406	0.10	0.93	1.00	0.06
Random platelets vs. Cross-matched platelets	74	3.26 (0.95–5.05)	4.00 (1.10–8.33)	−0.74	1,071	−1.53	0.13	0.66	0.17
Random platelets vs. Cross-matched platelets + IVIG	35	2.62 (0.73–6.39)	3.59 (1.17–7.77)	−0.97	285	−0.48	0.63	1.00	0.03
Cross-matched platelets vs. Cross-matched platelets + IVIG	26	3.10 (1.04–6.68)	3.88 (1.12–7.05)	−0.78	150	0.32	0.75	1.00	0.01

*p*_Holm_, *p*-values adjusted by Holm procedure for multiple testing; Cohen's *d_z_*: standardized effect size for within-patient paired comparisons; *x*_1_ = group 1, *x*_2_ = group 2.

These findings were confirmed by the mixed-effects analysis of all 710 episodes. The overall effect of transfusion strategy was not significant (*p* = 0.692; Figure 2A). As shown in Table 4A, adjusted 24 h CCI values were 3.81 (95% CI: 0.42–7.20) for random platelets alone, 3.69 (0.31–7.07) for random platelets plus IVIG, 4.22 (0.81–7.63) for cross-matched platelets alone and 4.49 (0.92–8.04) for cross-matched platelets plus IVIG. As shown in Table 4B, all adjusted pairwise differences were small (≤0.80) and non-significant (Tukey-adjusted *p* = 0.76–1.00), with confidence intervals excluding clinically meaningful differences. GEE models with robust standard errors yielded essentially identical estimates (all Holm-adjusted *p* = 1.00), as did all sensitivity analyses (Supplementary Table S3). As shown in Table 4C decomposition of the strategy effect showed that neither the within-patient nor the between-patient components differed significantly from zero, and the joint test of the contextual effects was not significant (*p* = 0.831). Among covariates, only transfusion sequence number was independently associated with 24 h CCI (*β* = 0.22 per additional transfusion, *p* < 0.001) (Supplementary Table S4).

**Figure 2:**
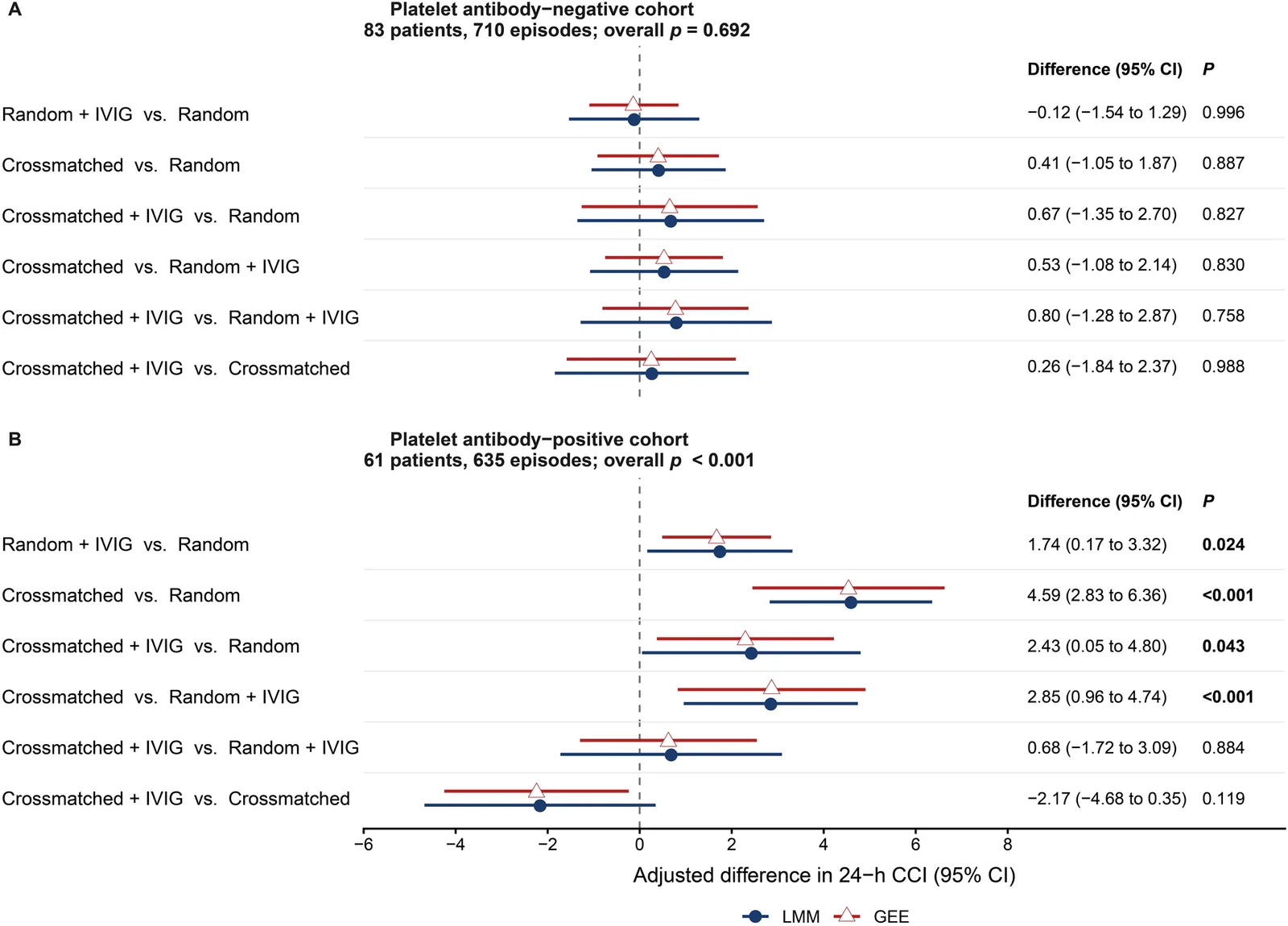
Adjusted between-strategy differences in 24-h corrected count increment (CCI). Forest plot of all six pairwise comparisons in the platelet antibody-negative **(A)** and antibody-positive **(B)** cohorts. Circles denote adjusted mean differences from linear mixed-effects models with a random intercept for patient (Tukey-adjusted *p* values shown); triangles denote population-averaged estimates from generalised estimating equations with an exchangeable working correlation and robust standard errors clustered on patient. Horizontal bars are 95% confidence intervals; the dashed line indicates no difference. Both models were adjusted for fever, transfusion sequence number, sex, age category, diagnostic group, treatment history, number of chemotherapy cycles, splenomegaly, GVHD, infection, DIC, and cumulative prior platelet transfusions. IVIG, intravenous immunoglobulin.

**Table 4A:** Covariate-adjusted marginal mean 24 h CCI by transfusion strategy.

Transfusion strategy	Adjusted 24 h CCI (95% CI)	SE	Adjusted 24 h CCI (95% CI)	SE
	Antibody-negative cohort		Antibody-positive cohort	
Random platelets	3.81 (0.42 to 7.20)	1.70	3.64 (−1.35 to 8.62)	2.50
Random platelets + IVIG	3.69 (0.31 to 7.07)	1.69	5.38 (0.40 to 10.36)	2.50
Cross-matched platelets	4.22 (0.81 to 7.63)	1.71	8.23 (3.17 to 13.28)	2.54
Cross-matched platelets + IVIG	4.49 (0.92 to 8.04)	1.79	6.06 (1.08 to 11.05)	2.50

Linear mixed-effects models with a random intercept for patient, adjusted for all covariates listed in Supplementary Table S4.

**Table 4B:** Adjusted between-strategy differences in 24 h CCI.

Comparison	LMM adjusted difference (95% CI)	Tukey-adjusted *p*	GEE difference (95% CI)	Holm-adjusted *p*
Antibody-negative cohort				
Random + IVIG vs. Random	−0.12 (−1.54 to 1.29)	0.996	−0.13 (−1.09 to 0.84)	1.000
Cross-matched vs. Random	0.41 (−1.05 to 1.87)	0.887	0.40 (−0.92 to 1.72)	1.000
Cross-matched + IVIG vs. Random	0.67 (−1.35 to 2.70)	0.827	0.65 (−1.26 to 2.56)	1.000
Cross-matched vs. Random + IVIG	0.53 (−1.08 to 2.14)	0.830	0.53 (−0.75 to 1.81)	1.000
Cross-matched + IVIG vs. Random + IVIG	0.80 (−1.28 to 2.87)	0.758	0.78 (−0.81 to 2.36)	1.000
Cross-matched + IVIG vs. Cross-matched	0.26 (−1.84 to 2.37)	0.988	0.25 (−1.59 to 2.09)	1.000
Antibody-positive cohort				
Random + IVIG vs. Random	1.74 (0.17 to 3.32)	0.024	1.67 (0.49 to 2.86)	0.028
Cross-matched vs. Random	4.59 (2.83 to 6.36)	<0.001	4.54 (2.45 to 6.63)	<0.001
Cross-matched + IVIG vs. Random	2.43 (0.05 to 4.80)	0.043	2.30 (0.37 to 4.22)	0.058
Cross-matched vs. Random + IVIG	2.85 (0.96 to 4.74)	<0.001	2.87 (0.83 to 4.91)	0.028
Cross-matched + IVIG vs. Random + IVIG	0.68 (−1.72 to 3.09)	0.884	0.62 (−1.30 to 2.55)	0.524
Cross-matched + IVIG vs. Cross-matched	−2.17 (−4.68 to 0.35)	0.119	−2.24 (−4.25 to −0.24)	0.058

LMM *p* values Tukey-adjusted; GEE *p* values Holm-adjusted. GEE: exchangeable working correlation, robust standard errors clustered on patient.

### 3.4 Efficacy of different transfusion strategies in antibody-positive patients with PTR

#### 3.4.1 Omnibus comparison across the four transfusion strategies

In antibody-positive patients, the 24 h CCI (mean ± SD) for random platelets was 0.83 ± 3.62, for cross-matched platelets 7.74 ± 9.02, for random platelets + IVIG 3.07 ± 5.52, and for cross-matched platelets + IVIG 4.72 ± 5.61. The Skillings-Mack test demonstrated a highly significant overall difference between the four strategies (*SM* = 23.73, *df* = 3, *p* < 0.001; Figure 1B).

#### 3.4.2 Pairwise comparisons between transfusion strategies

For each pairwise comparison of transfusion strategies, the analysis was restricted to patients receiving both interventions. Patient-level mean 24 h CCI values were compared using the Wilcoxon signed-rank test. Raw *p* values were adjusted via the Holm procedure to account for all six possible pairwise comparisons across the four strategies. Nevertheless, only four prespecified clinically relevant contrasts are presented in the results for this cohort. Specifically:

- Compared with random platelets alone, both cross-matched platelets and IVIG combined with random platelets were associated with improved 24 h CCI: all *p*_Holm_ < 0.001, with Cohen's *d_z_* = 0.69 (moderate effect) and 0.44 (small effect), respectively.
- Cross-matched platelets were associated with higher 24 h CCI relative to random plus IVIG: *p*_Holm_ = 0.019, Cohen's *d_z_* = 0.45 (small effect).
- Cross-matched platelets combined with IVIG showed no additional benefit compared with cross-matched platelets alone in this cohort: *p*_Holm_ = 0.444, Cohen's *d_z_* = 0.01 (small effect).

Accordingly, among patients with antibody-positive PTR, cross-matched platelets showed the most favorable response in this cohort. In addition high-dose IVIG combined with random platelets was also associated with improved 24-h CCI (Table 5).

**Table 5:** Pairwise comparisons between transfusion strategies in antibody-positive patients with PTR.

Paired group (*x*_1_ vs. *x*_2_)	*n*	Median (Q1–Q3)		Median difference	*Wilcoxon W*	*z*	*p_raw_*	*p*_Holm_	Cohen's *d_z_*
		*x*_1_	*x*_2_						
Random platelets vs. Random platelets + IVIG	59	0.00 (−1.34 to 1.53)	1.77 (0.00–3.22)	−1.77	328	−3.96	<0.001	<0.001	0.44
Random platelets vs. Cross-matched platelets	55	0.00 (−1.34 to 1.53)	5.60 (0.17–12.06)	−5.60	253	−4.33	<0.001	<0.001	0.69
Cross-matched platelets vs. Random platelets + IVIG	53	4.51 (0.00–12.16)	1.53 (0.00–3.19)	2.98	378	2.83	0.005	0.019	0.45
Cross-matched platelets vs. Cross-matched platelets + IVIG	20	2.34 (−0.10 to 6.69)	3.36 (0.51–7.64)	−1.02	84	−0.77	0.444	0.444	0.01

*p*_Holm_, *p*-values adjusted by Holm procedure for multiple testing; Cohen's *d_z_*: standardized effect size for within-patient paired comparisons; *x*_1_ = group 1, *x*_2_ = group 2.

The mixed-effects analysis of all 635 episodes confirmed and refined these results (*p* < 0.001; Figure 2B). As shown in Tables 4A, 4B, adjusted mean differences in 24 h CCI, relative to random platelets alone, were +1.74 (95% CI: 0.17–3.32; *p* = 0.024) for random platelets plus IVIG, +4.59 (2.83–6.36; *p* < 0.001) for cross-matched platelets alone and +2.43 (0.05–4.80; *p* = 0.043) for cross-matched platelets plus IVIG. Cross-matched platelets alone were also superior to random platelets plus IVIG (+2.85, 0.96–4.74; *p* < 0.001). In contrast, the addition of IVIG to cross-matched platelets conferred no additional benefit (cross-matched plus IVIG vs. cross-matched alone: −2.17, −4.68 to 0.35; *p* = 0.119), and cross-matched platelets plus IVIG did not differ from random platelets plus IVIG (+0.68, −1.72 to 3.09; *p* = 0.884). GEE models with robust standard errors produced closely similar estimates (+1.67, +4.54, +2.30, +2.87, −2.24 and +0.62, respectively; Holm-adjusted *p* = 0.028, <0.001, 0.058, 0.028, 0.058 and 0.524, respectively), and all sensitivity analyses, including rank-based and heteroscedastic models, reproduced the same pattern (Supplementary Table S3).

Because all patients received at least two strategies, the strategy effects could be decomposed into within- and between-patient components. As shown in Table 4C, all three within-patient contrasts remained significant (random plus IVIG +1.94, 95% CI: 0.69–3.19, *p* = 0.002; cross-matched alone +4.72, 3.34–6.09, *p* < 0.001; cross-matched plus IVIG +2.73, 0.87–4.59, *p* = 0.004), whereas none of the between-patient components was significant (*p* = 0.35–0.97), and the joint test of the contextual effects was non-significant (*p* = 0.559). The observed benefit of cross-matched platelets and of IVIG therefore derived from within-patient comparisons and was not attributable to systematic differences between patients selected for different strategies. Among covariates, GVHD (*β* = 5.65, *p* < 0.001) and transfusion sequence number (*β* = 0.18, *p* < 0.001) were positively associated, and the cumulative number of prior platelet transfusions negatively associated (*β* = −0.15, *p* = 0.037), with 24 h CCI (Supplementary Table S4).

**Table 4C:** Within- and between-patient decomposition of the strategy effect.

Comparison	Within-patient effect (95% CI)	*p*	Between-patient effect (95% CI)	*p*
Antibody-negative cohort				
Random + IVIG vs. Random	−0.14 (−1.25 to 0.96)	0.801	1.06 (−4.20 to 6.32)	0.695
Cross-matched vs. Random	0.41 (−0.72 to 1.55)	0.472	0.64 (−5.47 to 6.76)	0.837
Cross-matched + IVIG vs. Random	0.77 (−0.82 to 2.36)	0.342	−1.52 (−8.39 to 5.36)	0.667
Joint Wald test of contextual effects	Wald *χ*^2^ = 0.88, *p* = 0.831			
Antibody-positive cohort				
Random + IVIG vs. Random	1.94 (0.69 to 3.19)	0.002	0.10 (−4.86 to 5.07)	0.968
Cross-matched vs. Random	4.72 (3.34 to 6.09)	<0.001	3.18 (−3.36 to 9.72)	0.347
Cross-matched + IVIG vs. Random	2.73 (0.87 to 4.59)	0.004	−1.35 (−9.93 to 7.22)	0.759
Joint Wald test of contextual effects	Wald *χ*^2^ = 2.07, *p* = 0.559			

Obtained by cluster-mean centring within a linear mixed-effects model.

Notably, accounting for within-patient correlation altered the results in both directions relative to a naive episode-level analysis: the benefit of adding IVIG to random platelets, which was not statistically significant when episodes were treated as independent (Tukey-adjusted *p* = 0.224), became significant in the mixed model (*p* = 0.024), because this contrast is largely informed by within-patient comparisons (Supplementary Table S3B); conversely, the apparent advantage of cross-matched platelets plus IVIG over random platelets alone was attenuated (*p* = 0.005 to *p* = 0.043). This illustrates that ignoring clustering may both conceal and exaggerate treatment effects, and supports the use of models that explicitly represent the hierarchical structure of the data.

## 4 Discussion

This retrospective cohort study examined factors linked to platelet antibody development in hematologic patients with PTR and assessed the clinical efficacy of cross-matched platelets and IVIG. The findings inform transfusion strategies for antibody-negative and antibody-positive PTR patients, help identify who benefits from cross-matched platelets or IVIG, and support rational allocation of cross-matched platelets.

A methodological strength of the present study is that transfusion strategy varied within patients in the entire cohort, so that every patient acted as his or her own control. This allowed the strategy effect to be decomposed into within- and between-patient components; the finding that the effects were driven entirely by within-patient contrasts, with non-significant contextual effects, argues against confounding by indication—an important consideration in a retrospective study in which cross-matched platelets and IVIG are preferentially allocated to patients perceived as refractory. Consistency of the estimates across mixed-effects models, population-averaged GEE models, non-parametric incomplete-block tests and self-matched paired comparisons further supports the robustness of the conclusions.

Platelet antibodies were detected in 42.36% of 144 PTR patients—higher than the 10%–25% reported by Cohn et al. (21) and the 18% reported by Legler et al. (5). This difference likely reflects our study population: all had hematologic disorders requiring repeated platelet transfusions during chemotherapy-induced marrow suppression or after hematopoietic stem cell transplantation. Because current practice ensures only ABO and Rh matching without routine HLA or HPA genotyping, transfusion of mismatched platelets readily triggers alloimmunization.

Univariate analysis identified sex, age, obstetric history, diagnosis, and chemotherapy cycles as factors associated with platelet antibody development. After multivariate adjustment, female sex remained the strongest independent predictor (OR: 4.18, 95% CI: 2.25–8.24). Several mechanisms explain this disparity: pregnancy-related alloimmunization (including unrecognized miscarriages), estrogen-enhanced B-cell survival and antibody production, and X-linked immune-regulatory genes (e.g., TLR7, FOXP3) with differential expression between sexes. Women with a history of pregnancy are at particular risk because maternal–fetal antigen incompatibility exposes them to paternal HLA/HPA antigens. In a blood donor study, HLA antibody positivity was 17.3% in women; among those with prior pregnancy, the rate reached 24.4%, compared with 1.7% in transfused men and 1.0% in non-transfused men (22). Positivity rose with the number of pregnancies: 1.7% (0), 11.2% (1), 22.5% (2), 27.5% (3), and 32.2% (≥4) (22).

Older age also increased risk, likely reflecting longer disease duration and greater cumulative exposure to immunogenic stimuli (transfusions, pregnancy). We found no association between cumulative platelet transfusions and antibody development, consistent with Dutcher (23) and Schiffer (24), who reported no dose–response relationship. In our study, many patients receiving ≥6 transfusions had complex clinical courses with nonimmune factors, which may have confounded the analysis. A prospective study tracking the same cohort from first transfusion to antibody appearance would better address this question.

The risk of antibody development decreased with more chemotherapy cycles, suggesting that immune-mediated PTR occurs mostly during the first cycle. Chemotherapy suppresses immune function, reducing B lymphocytes and plasma cells and thus lowering antibody production. Additional cycles also increase the likelihood of nonimmune factors such as infection, fever, sepsis, and DIC. Chemotherapy-induced thrombocytopenia may arise from vascular endothelial damage, leading to platelet adhesion and consumption; cumulative toxicity suppresses megakaryopoiesis and directly destroys transfused platelets (25).

Univariate analysis identified MDS and AML/CML as risk factors, but multivariate analysis did not confirm this. Multivariate analyses in the present study suggested a potential association between lymphoma and antibody production. Nevertheless, this finding is constrained by the extremely small subgroup sample size, wide confidence intervals and insufficient statistical power. The true effect of lymphoma on antibody production should therefore be evaluated with caution. Further multicenter, large-sample prospective clinical studies are required to validate this observation.

Although age, chemotherapy cycles, and obstetric history showed significant associations in univariate analysis, these factors were no longer statistically significant following multivariate adjustment. This finding is likely attributable to the limited sample size and residual selection bias.

No consensus exists on a gold standard test for diagnosing immune PTR (14). Based on our findings, we suggest platelet antibody screening for patients with a history of pregnancy, those with MDS or AML/CML, and patients receiving their first chemotherapy cycle, which may facilitate early identification of patients at high risk of alloimmunization.

Among patients with antibody-negative PTR, cross-matched platelets and IVIG demonstrated no additional benefit in this cohort. Some clinicians routinely administer cross-matched platelets for antibody-negative PTR patients; however, our findings do not lend support to this clinical practice. For these patients, clinicians may focus on identifying and managing non-immune factors such as splenomegaly, DIC, fever, infection, persistent bleeding, and antibiotic use.

This study provides real-world evidence to guide platelet transfusion strategy selection for antibody-negative patients with PTR. Studies that exclude patients with non-immune factors overestimate the efficacy of cross-matched platelets (26).

In antibody-positive PTR, cross-matched platelets yielded the most favorable response in our cohort. This agrees with Sinwatcharaphirom (27), Wiita (28), and Kaur (29). Although large blood centers may maintain HLA/HPA-typed donor registries, such resources are not widely available. Serologic cross-matching using SPRCA provides a rapid, effective, and broadly applicable alternative.

Among patients with antibody-positive PTR, IVIG combined with random platelets was associated with improved 24 h CCI. IVIG enhances platelet transfusion responses by binding antigens, activating complement, blocking Fc*γ* receptors on mononuclear phagocytes, and reducing platelet destruction (12, 17, 30). However, in this cohort, cross-matched platelet transfusion yielded superior 24 h CCI values relative to random platelet transfusion combined with high-dose IVIG (*p*_Holm_ = 0.019). Kickler et al. (31) also advised against using IVIG in place of compatible platelets, noting that IVIG improves 1-h but not 24-h CCI. Nevertheless, in emergencies when cross-matched platelets are unavailable, high-dose IVIG combined with random platelets may be preferentially considered.

Notably, cross-matched platelets combined with IVIG showed no additional benefit compared with cross-matched platelets alone in this cohort (*p*_Holm_ = 0.444). Several potential mechanisms may account for this absence of additive therapeutic effect. Cross-matched platelets already circumvent the major pathway of immune destruction—HLA or HPA antibody-mediated clearance. IVIG's primary mechanism in alloimmune thrombocytopenia is Fc*γ* receptor blockade on splenic macrophages; once incompatible antigens are avoided by cross-matching, the target for IVIG disappears. IVIG may also paradoxically suppress endogenous platelet production or interfere with platelet survival, although this interpretation remains speculative.

In addition to these mechanistic considerations, the observed findings may partly stem from study-specific limitations. On the one hand, heterogeneous IVIG dosing in our real-world cohort represents an important confounding factor; on the other hand, inter-individual variability in patients' immune status and underlying disease severity could also contribute to these outcomes. This is a retrospective real-world study, in which IVIG administration was highly dependent on individual clinical decisions by attending physicians, with doses ranging from 5 g to 25 g. The absence of a weight-based standardized dosing regimen (e.g., 0.4 g/kg) and lack of fixed administration timing represent key limitations of the present study. Variable therapeutic exposure arising from this heterogeneity may mask the genuine clinical benefit of IVIG. This limitation weakens the evidentiary strength of our clinical recommendation against combining cross-matched platelets with IVIG. Future prospective controlled studies adopting weight-standardized dosing and fixed administration timing are warranted to clarify the true therapeutic effect of IVIG in this setting.

Some patients receiving both cross-matched platelets and IVIG were critically ill, and IVIG may have been given for infection control or B-cell depletion rather than to enhance platelet increments. As a retrospective single-center real-world study, the administration of cross-matched platelet transfusion and IVIG treatment relied on individualized clinical decisions by attending physicians. Specifically, patients with critical illness and higher inflammatory burden were more likely to receive IVIG intervention, while those with refractory antibody positivity tended to be prescribed cross-matched platelet transfusion. This indication bias may impact the comparative efficacy results of different treatment regimens. Although this study adopted multi-covariate adjustment and stratification (antibody negative/positive) to mitigate bias, the retrospective single-center design still cannot completely eliminate the residual bias caused by individual baseline differences among patients. Therefore, the conclusions of this study need to be further verified by multi-center prospective studies.

Our study demonstrated that female sex is the strongest independent predictor of platelet antibody development, with direct clinical utility. We recommend early platelet antibody screening for female PTR patients, especially those of childbearing age or with a history of pregnancy, preferably during the first chemotherapy cycle or at at the initial diagnosis of PTR. Such targeted screening facilitates the early recognition of patients who may benefit from cross-matched platelet transfusion. These findings support a stratified and personalized approach to PTR management, which may help reduce unnecessary use of cross-matched platelet products and the associated resource use.

PTR remains a multifactorial clinical challenge. Some patients with immune-mediated PTR exhibit suboptimal responses even to cross-matched platelets transfusion, potentially owing to unidentified antibodies or coexisting non-immune factors. Prompt post-transfusion efficacy assessment and individualized clinical workup are therefore essential for optimal patient management.

## 5 Conclusions

Among hematologic PTR patients, platelet antibody formation links to sex, obstetric history, age, diagnosis and chemotherapy cycles; sex is the sole independent predictor. Among patients with antibody-negative PTR, cross-matched platelets and IVIG demonstrated no additional benefit in this cohort. For antibody-positive PTR, cross-matched platelets yielded the most favorable response in our cohort; when unavailable, high-dose IVIG combined with random platelets may be preferentially considered. These findings support a stratified and personalized approach to PTR management. These comparative treatment findings require prospective validation.

## Data Availability

The data analyzed in this study is subject to the following licenses/restrictions: The datasets presented in this article are not readily available because they contain identifiable information from human subjects that cannot be shared in open access repositories for legal and ethical reasons. Anonymized data will be shared upon request from any qualified investigator. Requests to access these datasets should be directed to Huayou Zhou, zohoyo@smu.edu.cn.
