# Supplementary material for: Personalized management of platelet transfusion refractoriness: risk factors, cross-matching, and IVIG strategies
Source: Front Med (Lausanne). 2026 Sep 18;13:1923303. doi: 10.3389/fmed.2026.1923303 (PMC13630665; doi:10.3389/fmed.2026.1923303)
Supplement: Supplementary file 1 [file Datasheet1.pdf]

Supplementary Tables

Supplementary Table S1. Data structure and within-patient clustering.

Supplementary Table S2A. Shared patients, antibody-negative cohort **and Table S2B.** Shared patients, antibody-positive cohort.

Supplementary Table S3A. Omnibus tests **and Table S3B.** Eleven analytical approaches: difference in 24-h CCI (adjusted P).

Supplementary Table S4. Complete fixed-effects estimates.

Supplementary Table S1. Data structure and within-patient clustering.			
**Section**	**Variable**	**Antibody-negative (n = 83)**	**Antibody-positive (n = 61)**
Cohort size and clustering	Patients, n	83	61
Cohort size and clustering	Transfusion episodes, n	710	635
Cohort size and clustering	Episodes per patient, mean	8.55	10.41
Cohort size and clustering	Episodes per patient, median (IQR)	7 (4-12)	9 (6-13)
Cohort size and clustering	Episodes per patient, range	2-27	3-39
Number of strategies received per patient	Received 1 strategy, n (%)	0 (0.0)	0 (0.0)
Number of strategies received per patient	Received 2 strategies, n (%)	5 (6.0)	1 (1.6)
Number of strategies received per patient	Received 3 strategies, n (%)	53 (63.9)	41 (67.2)
Number of strategies received per patient	Received 4 strategies, n (%)	25 (30.1)	19 (31.1)
Variance components and design effect	Between-patient variance, tau^2	6.912	5.552
Variance components and design effect	Residual variance, sigma^2	30.251	39.299
Variance components and design effect	Intraclass correlation coefficient (ICC)	0.186	0.124
Variance components and design effect	LRT for patient random intercept, chi^2 (df = 1)	32.06	13.38
Variance components and design effect	P value, LRT	<0.001	<0.001
Variance components and design effect	Mean cluster size, episodes	8.55	10.41
Variance components and design effect	Conventional design effect, 1 + (m-1)*ICC	2.41	2.17
Variance components and design effect	Effective sample size	295	293
Variance components and design effect	SE inflation factor if clustering ignored, sqrt(Deff)	1.55	1.47
Episodes and 24-h CCI by transfusion strategy	Random platelets - episodes, n	290	259
Episodes and 24-h CCI by transfusion strategy	Random platelets + IVIG - episodes, n	187	180
Episodes and 24-h CCI by transfusion strategy	Crossmatched platelets - episodes, n	162	128
Episodes and 24-h CCI by transfusion strategy	Crossmatched platelets + IVIG - episodes, n	71	68
Episodes and 24-h CCI by transfusion strategy	Random platelets - patients exposed, n	83	61
Episodes and 24-h CCI by transfusion strategy	Random platelets + IVIG - patients exposed, n	77	59
Episodes and 24-h CCI by transfusion strategy	Crossmatched platelets - patients exposed, n	74	55
Episodes and 24-h CCI by transfusion strategy	Crossmatched platelets + IVIG - patients exposed, n	35	26

Supplementary Table S1. Data structure and within-patient clustering.

Section	Variable	Antibody-negative (n = 83)	Antibody-positive (n = 61)
Episodes and 24-h CCI by transfusion strategy	Random platelets - episode-level CCI, mean (SD)	3.83 (5.88)	1.61 (5.77)
Episodes and 24-h CCI by transfusion strategy	Random platelets + IVIG - episode-level CCI, mean (SD)	3.50 (5.48)	2.82 (6.19)
Episodes and 24-h CCI by transfusion strategy	Crossmatched platelets - episode-level CCI, mean (SD)	4.55 (6.68)	6.16 (8.27)
Episodes and 24-h CCI by transfusion strategy	Crossmatched platelets + IVIG - episode-level CCI, mean (SD)	4.47 (6.85)	4.54 (6.05)
Episodes and 24-h CCI by transfusion strategy	Random platelets - patient-level mean CCI, mean (SD)	4.15 (5.56)	0.83 (3.62)
Episodes and 24-h CCI by transfusion strategy	Random platelets + IVIG - patient-level mean CCI, mean (SD)	3.75 (4.36)	3.07 (5.52)
Episodes and 24-h CCI by transfusion strategy	Crossmatched platelets - patient-level mean CCI, mean (SD)	5.33 (5.95)	7.74 (9.02)
Episodes and 24-h CCI by transfusion strategy	Crossmatched platelets + IVIG - patient-level mean CCI, mean (SD)	4.80 (6.18)	4.72 (5.61)
Episodes and 24-h CCI by transfusion strategy	Random platelets - patient-level mean CCI, median (IQR)	3.20 (0.84 to 5.38)	0.00 (-1.34 to 1.86)
Episodes and 24-h CCI by transfusion strategy	Random platelets + IVIG - patient-level mean CCI, median (IQR)	2.72 (1.09 to 5.40)	1.77 (0.00 to 3.22)
Episodes and 24-h CCI by transfusion strategy	Crossmatched platelets - patient-level mean CCI, median (IQR)	4.00 (1.10 to 8.33)	5.60 (0.17 to 12.06)
Episodes and 24-h CCI by transfusion strategy	Crossmatched platelets + IVIG - patient-level mean CCI, median (IQR)	3.59 (1.17 to 7.77)	3.36 (0.84 to 8.10)

See Excel version for full footnote.

Supplementary Table S2A. Shared patients, antibody-negative cohort.

Strategy	Random platelets	Random platelets + IVIG	Crossmatched platelets	Crossmatched platelets + IVIG
Random platelets	83			
Random platelets + IVIG	77	77		
Crossmatched platelets	74	69	74	
Crossmatched platelets + IVIG	35	33	26	35

Diagonal: patients exposed; off-diagonal: patients exposed to both.

Supplementary Table S2B. Shared patients, antibody-positive cohort.

Strategy	Random platelets	Random platelets + IVIG	Crossmatched platelets	Crossmatched platelets + IVIG
Random platelets	61			
Random platelets + IVIG	59	59		
Crossmatched platelets	55	53	55	
Crossmatched platelets + IVIG	26	25	20	26

Diagonal: patients exposed; off-diagonal: patients exposed to both.

**Supplementary Table S3A. Omnibus tests.**		
**Omnibus test across the four strategies**	**Antibody-negative**	**Antibody-positive**
Naive episode-level one-way ANOVA (reference only)	F(3,706) = 1.08, P = 0.359	F(3,631) = 15.22, P < 0.001
Naive episode-level Kruskal-Wallis (reference only)	chi^2(3) = 2.43, P = 0.488	chi^2(3) = 50.51, P < 0.001
ANOVA on patient-level means, unpaired (reference only)	F(3,265) = 1.20, P = 0.312	F(3,197) = 12.27, P < 0.001
Kruskal-Wallis on patient-level means, unpaired (reference only)	chi^2(3) = 2.56, P = 0.464	chi^2(3) = 31.90, P < 0.001
Skillings-Mack test (incomplete block, patient-level means)	chi^2(3) = 4.16, P = 0.244	chi^2(3) = 23.73, P < 0.001
Linear mixed-effects model, type III F test (primary)	F(3,664) = 0.49, P = 0.692	F(3,608) = 15.17, P < 0.001

See Excel version for full footnote.

Supplementary Table S3B. Eleven analytical approaches: difference in 24-h CCI (adjusted P).

**Analytical approach**	**Random + IVIG vs. Random**	**Crossmatched vs. Random**	**Crossmatched + IVIG vs. Random**	**Crossmatched vs. Random + IVIG**	**Crossmatched + IVIG vs. Random + IVIG**	**Crossmatched + IVIG vs. Crossmatched**
1. Naive episode-level ANOVA + Tukey (reference)	-0.33 (0.938)	0.72 (0.624)	0.64 (0.857)	1.05 (0.374)	0.97 (0.661)	-0.08 (1.000)
2. Self-matched Wilcoxon on patient means (Holm)	-0.33 (1.000)	1.31 (0.655)	0.24 (1.000)	1.40 (0.655)	0.65 (1.000)	0.04 (1.000)
3. Incomplete-block LMM on patient means	-0.38 (0.963)	1.20 (0.433)	0.59 (0.940)	1.57 (0.212)	0.96 (0.789)	-0.61 (0.938)
4. LMM, unadjusted	-0.28 (0.960)	0.56 (0.760)	0.88 (0.680)	0.83 (0.542)	1.16 (0.481)	0.32 (0.980)
5. LMM, adjusted [PRIMARY]	-0.12 (0.996)	0.41 (0.887)	0.68 (0.827)	0.53 (0.830)	0.80 (0.758)	0.26 (0.988)
6. GEE, exchangeable, robust SE	-0.12 (1.000)	0.40 (1.000)	0.65 (1.000)	0.53 (1.000)	0.78 (1.000)	0.25 (1.000)
7. GEE, independence, robust SE	-0.20 (1.000)	0.24 (1.000)	0.43 (1.000)	0.44 (1.000)	0.62 (1.000)	0.19 (1.000)
8. LMM, first episode of each strategy only	0.63 (0.902)	1.63 (0.315)	1.86 (0.425)	1.00 (0.727)	1.23 (0.751)	0.23 (0.998)
9. LMM, excluding patients above the 95th percentile of episodes	-0.02 (1.000)	0.33 (0.951)	0.71 (0.843)	0.35 (0.956)	0.74 (0.842)	0.38 (0.975)
10. Rank-based (van der Waerden) LMM*	0.02 (0.994)	0.05 (0.938)	0.14 (0.682)	0.03 (0.991)	0.12 (0.799)	0.09 (0.908)
11. LMM with heteroscedastic residual variance	-0.12 (0.995)	0.40 (0.916)	0.60 (0.886)	0.52 (0.847)	0.72 (0.816)	0.20 (0.996)
1. Naive episode-level ANOVA + Tukey (reference)	1.21 (0.224)	4.55 (<0.001)	2.93 (0.005)	3.34 (<0.001)	1.72 (0.245)	-1.62 (0.343)
2. Self-matched Wilcoxon on patient	2.50 (<0.001)	6.96 (<0.001)	3.48 (0.019)	4.56 (0.019)	1.94 (0.346)	0.04 (0.452)

Supplementary Table S3B. Eleven analytical approaches: difference in 24-h CCI (adjusted P).						
**Analytical approach**	**Random + IVIG vs. Random**	**Crossmatched vs. Random**	**Crossmatched + IVIG vs. Random**	**Crossmatched vs. Random + IVIG**	**Crossmatched + IVIG vs. Random + IVIG**	**Crossmatched + IVIG vs. Crossmatched**
means (Holm)						
3. Incomplete-block LMM on patient means	2.29 (0.140)	6.92 (<0.001)	4.20 (0.016)	4.63 (<0.001)	1.90 (0.529)	-2.72 (0.229)
4. LMM, unadjusted	1.60 (0.048)	5.06 (<0.001)	2.78 (0.013)	3.46 (<0.001)	1.17 (0.584)	-2.28 (0.086)
5. LMM, adjusted [PRIMARY]	1.74 (0.024)	4.59 (<0.001)	2.43 (0.043)	2.85 (<0.001)	0.68 (0.884)	-2.17 (0.119)
6. GEE, exchangeable, robust SE	1.67 (0.028)	4.54 (<0.001)	2.30 (0.058)	2.87 (0.028)	0.62 (0.524)	-2.24 (0.058)
7. GEE, independence, robust SE	1.64 (0.025)	4.51 (<0.001)	2.22 (0.067)	2.87 (0.025)	0.57 (0.553)	-2.29 (0.067)
8. LMM, first episode of each strategy only	3.14 (0.064)	7.49 (<0.001)	5.76 (0.012)	4.35 (0.010)	2.62 (0.422)	-1.73 (0.713)
9. LMM, excluding patients above the 95th percentile of episodes	1.62 (0.058)	4.75 (<0.001)	2.94 (0.047)	3.13 (<0.001)	1.32 (0.656)	-1.81 (0.436)
10. Rank-based (van der Waerden) LMM*	0.38 (<0.001)	0.66 (<0.001)	0.45 (0.007)	0.28 (0.055)	0.07 (0.956)	-0.21 (0.488)
11. LMM with heteroscedastic residual variance	1.78 (0.010)	4.53 (<0.001)	2.10 (0.058)	2.75 (0.008)	0.32 (0.982)	-2.43 (0.083)

See Excel version for full footnote.

Supplementary Table S4. Complete fixed-effects estimates.						
**Term**	**beta (SE)**	**df**	**P**	**beta (SE)**	**df**	**P**
	Antibody-negative cohort			Antibody-positive cohort		
(Intercept)	5.40 (1.89)	52.9	0.006	2.65 (1.98)	46.8	0.188
Transfusion strategy: Random + IVIG (vs. Random)	-0.12 (0.55)	672.5	0.826	1.74 (0.61)	610.2	0.005
Transfusion strategy: Crossmatched (vs. Random)	0.41 (0.57)	663.5	0.468	4.59 (0.69)	609.6	<0.001
Transfusion strategy: Crossmatched + IVIG (vs. Random)	0.67 (0.79)	677.5	0.392	2.43 (0.92)	612.0	0.009
Fever >=38 C (yes vs. no)	-0.41 (0.77)	675.8	0.596	-1.40 (1.05)	599.4	0.182
Splenomegaly (yes vs. no)	-1.94 (1.33)	61.0	0.148	-0.85 (1.18)	38.6	0.476
GVHD (yes vs. no)	1.15 (1.34)	62.8	0.392	5.65 (1.48)	31.7	<0.001
Infection (yes vs. no)	0.99 (1.56)	63.4	0.526	0.72 (1.31)	40.3	0.584
DIC (yes vs. no)	-1.43 (2.36)	59.1	0.546	-1.50 (3.99)	91.9	0.708
Sex (female vs. male)	0.35 (0.84)	51.0	0.674	0.48 (1.04)	43.3	0.649
Age group 2 (vs. group 1)	-1.37 (1.14)	59.1	0.235	0.50 (1.21)	39.3	0.685
Age group 3 (vs. group 1)	-2.23 (1.09)	60.7	0.045	-0.43 (0.88)	44.1	0.627
Diagnostic group 2 (vs. group 1)	0.28 (1.64)	52.4	0.864	0.22 (1.35)	45.6	0.870
Diagnostic group 3 (vs. group 1)	2.52 (1.88)	56.8	0.185	-0.62 (1.74)	49.2	0.725
Diagnostic group 4 (vs. group 1)	0.29 (2.57)	57.7	0.911	0.46 (2.95)	31.4	0.876
Diagnostic group 5 (vs. group 1)	-1.84 (2.08)	53.8	0.380	2.42 (3.03)	82.7	0.426
Treatment history 2 (vs. group 1)	0.93 (1.41)	58.4	0.513	1.57 (1.36)	42.6	0.254
Treatment history 3 (vs. group 1)	1.26 (1.16)	59.7	0.283	0.89 (1.30)	48.0	0.499
Chemotherapy cycles group 2 (vs. group 1)	-2.58 (1.34)	61.7	0.059	0.34 (1.19)	45.7	0.774
Chemotherapy cycles group 3 (vs. group 1)	-2.24 (1.27)	53.6	0.084	-1.12 (1.15)	42.6	0.336
Non-immune factors (yes vs. no)	1.55 (1.65)	65.9	0.351	-1.49 (1.32)	39.8	0.267
Cumulative prior platelet transfusions (per unit)	-0.08 (0.05)	50.2	0.101	-0.15 (0.07)	42.4	0.037
Transfusion sequence number (per episode)	0.22 (0.05)	687.0	<0.001	0.18 (0.05)	607.4	<0.001

See Excel version for full footnote.
